# Maximum-scoring path sets on pangenome graphs of constant treewidth

**DOI:** 10.3389/fbinf.2024.1391086

**Published:** 2024-07-01

**Authors:** Broňa Brejová, Travis Gagie, Eva Herencsárová, Tomáš Vinař

**Affiliations:** ^1^ Department of Computer Science, Faculty of Mathematics, Physics and Informatics, Comenius University in Bratislava, Bratislava, Slovakia; ^2^ Faculty of Computer Science, Dalhousie University, Halifax, NS, Canada; ^3^ Department of Applied Informatics, Faculty of Mathematics, Physics and Informatics, Comenius University in Bratislava, Bratislava, Slovakia

**Keywords:** treewidth, dynamic programming, weighted paths, pangenomics, elastic degenerate strings

## Abstract

We generalize a problem of finding maximum-scoring segment sets, previously studied by Csűrös (IEEE/ACM Transactions on Computational Biology and Bioinformatics, 2004, 1, 139–150), from sequences to graphs. Namely, given a vertex-weighted graph *G* and a non-negative startup penalty *c*, we can find a set of vertex-disjoint paths in *G* with maximum total score when each path’s score is its vertices’ total weight minus *c*. We call this new problem *maximum-scoring path sets* (MSPS). We present an algorithm that has a linear-time complexity for graphs with a constant treewidth. Generalization from sequences to graphs allows the algorithm to be used on pangenome graphs representing several related genomes and can be seen as a common abstraction for several biological problems on pangenomes, including searching for CpG islands, ChIP-seq data analysis, analysis of region enrichment for functional elements, or simple chaining problems.

## 1 Introduction

We study the maximum-scoring path set (MSPS) problem where the input is a directed or undirected graph with weighted vertices and a non-negative startup penalty *c*. The goal is to find a set of vertex-disjoint paths with maximum total score, where the score of each path is the sum of its vertex weights minus the cost *c*. This problem is a generalization of the maximum segment sum problem, where the input is a sequence of weights and penalty *c*, and we are looking for a set of disjoints segments with the maximum total score where again the score of each segment is the sum of weights minus the penalty. This simpler problem on sequences was studied extensively by Csűrös (2004), who provided a simple linear-time algorithm and connections to various statistical models of biological sequence segmentation. Csűrös (2004) as well as other authors (Bengtsson and Chen, 2007; Gawrychowski and Nicholson, 2015) also studied a variant of this problem where instead of startup cost *c* we are looking for the solution with a fixed number *k* of segments.

In this work we show that the problem can be solved in linear time not only for sequences, which are simple path graphs, but also on graphs with the treewidth bounded by a constant. Graphs with a constant treewidth are an important generalization of trees. Many problems can be solved by more efficient algorithms on constant treewidth graphs than on general graphs (Arnborg et al., 1991; Bodlaender, 1997). By extending the linear-time algorithm from sequences to graphs, it can be now applied to pangenome graphs representing sets of related sequences. We describe biological motivation of the problem in more detail in Section 2. Graphs of bounded treewidth were previously studied in bioinformatics, for example, for phylogenetic networks (Scornavacca and Weller, 2022) and RNA structures (Marchand et al., 2022), but as far as we know, they were not previously considered in the context of pangenomics. Gómez and Wakabayashi (2020) solved some related problems but their proofs are inaccessible to many bioinformaticians, as they rely on Courcelle’s theorem that every graph property definable in monadic second order logic can be decided in linear time on graphs with bounded treewidth. Among the problems they studied, the one closest to our goal is the MaxWNtPc problem, which seeks to cover all vertices of an undirected graph with weighted edges by non-trivial vertex-disjoint paths with the maximum total weight. It is not obvious how to reduce MSPS to this problem, and also the authors consider only undirected graphs.

## 2 Motivation

Many computational or high-throughput wet-lab analyses of genomes identify chromosome locations that have some biological function or property. We may then want to search for dense clusters of such significant locations. The simplest examples are based on sequence content (Csűrös, 2004; Deaton and Bird, 2011), such as looking for GC-rich regions (regions with a high density of bases C and G) or CpG islands (regions with a high density of C followed by G). Such areas are often associated with functional elements such as genes or regulatory regions (Li et al., 2002; Deaton and Bird, 2011). A more complex example is search for clusters of nearby binding sites of transcription factors (Wu et al., 2022); potential binding sites can be identified computationally based on occurrence of known sequence motifs or experimentally by techniques such as chromatin immunoprecipitation followed by sequencing (ChIP-seq). We can also identify positions of sequence differences among related species or individuals within a single species. Then we can look for regions with a high density of such mutations, which can arise, for example, from horizontal sequence transfer (Croucher et al., 2015). Conversely, regions with unusually low number of such mutations are conserved in evolution by purifying selection and may represent important functional elements (Stojanovic et al., 1999).

All of these examples involve identifying individual positions of a genomic sequence with some biological property and then looking for clusters of such positions located close together. Such clustering problems can be formulated in many ways (Kulldorff, 1999; Li et al., 2002; Coppe et al., 2006; Ferrari et al., 2011; Croucher et al., 2015; He et al., 2019), but we will concentrate on the segmentation approach of Csűrös (2004), where we assign a positive score to each identified position of interest and a negative score to all other positions of the genome and then find non-overlapping high-scoring segments in the resulting sequence of scores. Startup penalty *c* charged for each segment in the solution controls the number of the resulting segments. A small value of *c* yields many short segments, while at higher values some nearby segments may be joined together and weaker segments may be omitted. Csűrös (2004) shows that several statistical approaches to defining clusters of positions can be expressed by an appropriate choice of position scores and penalty *c*.

In this work, we extend the segmentation approach from sequences to sequence graphs (Computational Pan-Genomics Consortium, 2018). In a sequence graph, vertices represent sequence fragments and edges possible adjacencies of these fragments. Chromosomes then form paths or walks in these graphs. To find a segmentation, we assign scores to individual vertices, when necessary splitting a vertex into a path so that each vertex of the path represents a single nucleotide. Instead of non-overlapping segments of the input sequence we seek vertex-disjoint paths in the graph.

The notion of a sequence graph has been used in many studies that generalize genomic analyses from a single sequence to an ensemble of sequences, including structures such as splicing graphs (Heber et al., 2002), A-Bruijn graphs (Pevzner et al., 2004), Enredo graphs (Paten et al., 2008), cactus graphs (Paten et al., 2011), colored de Bruijn graphs (Iqbal et al., 2012), variation graphs (Garrison et al., 2018), and reference pangenome graphs (Li et al., 2020). The field of pangenomics aims to shift computational analyses from using a single reference genome for each species to using a collection of genomes of different individuals representing genetic diversity of the species (a pangenome). This approach was shown to reduce biases caused by a single reference (Garrison et al., 2018), but requires us to adapt existing algorithms developed for a single sequence to work on sequence sets or pangenome graphs. Here we contribute to this effort by studying the sequence segmentation problem in the graph context.

Several existing pangenomic methods are related to our goal. Grytten et al. (2019) analyze ChIP-seq data in the context of a pangenome graph. First they identify positions with significantly increased read coverage and then use simple heuristics to connect them into longer paths with high density of such positions representing likely areas where the studied molecule binds DNA. This heuristic process could be replaced by our segmentation approach. Chang et al. (2020) study the problem of mapping reads to a pangenome graph by first identifying short seed matches between the read and the sequences represented by the graph. Then they look for clusters of nearby seed matches which could correspond to regions where the read actually aligns. They define clusters as connected components in an auxiliary graph where two seeds are connected by an edge if their distance in the pangenome is below a certain threshold. An alternative would be to assign suitable positive scores to seed positions and negative scores elsewhere and look for high-scoring segments using our approach. Several authors have studied a related but more complex co-linear chaining problem (Mäkinen et al., 2019; Li et al., 2020; Chandra and Jain, 2023; Ma et al., 2023; Rizzo et al., 2023; Rajput et al., 2024), where we look for a walk containing many seeds, but these seeds should occur in the same order along the walk and in the input read. Algorithms for the chaining problem seek to find a single best walk, perhaps with additional suboptimal walks returned as well, but no care is taken to find the globally optimal combination of disjoint walks or paths.

Our algorithm works efficiently only on graphs with a small treewidth with special cases, such as directed series-parallel graphs, admitting particularly simple variants of the algorithm. There are many different ways of defining and building pangenome graphs, some of them yielding graphs of small treewidth. One popular representation is an *elastic degenerate string* (EDS) (Iliopoulos et al., 2021). As we show in Section 3.3, an EDS can be converted to a directed series-parallel graph.

Another class of pangenome graphs considered in the literature are general directed acyclic graphs (DAGs). While some algorithms run quickly on arbitrary DAGs (Grytten et al., 2019), others run quickly only on graphs which have a small path cover, which is in this case defined as the smallest number of potentially overlapping paths that cover each vertex at least once (Mäkinen et al., 2019; Chandra and Jain, 2023; Ma et al., 2023; Rizzo et al., 2023). Note that this measure is non-increasing upon addition of new edges, whereas treewidth is non-decreasing. A more relevant measure is the arc-width, introduced by Tomescu et al. (2015), which is defined as the smallest number of paths needed to cover all arcs of a directed graph. A pangenome DAG constructed from *k* distinct sequences can be covered by *k* paths, one for each sequence, and thus it will have the arc-width at most *k*. As we discuss in Section 4, a DAG with arc-width *k* has treewidth (and pathwidth) at most *k*, and thus acyclic pangenomes assembled from a small number of genomes are suitable inputs to our algorithm. Nonetheless, it should be noted that pangenome graphs containing cycles are also used (Li et al., 2020), and these can be handled by our algorithm only if their treewidth is small. In a preliminary version of this work, we studied DAGs of small pathwidth (Herencsárová and Brejová, 2023), which is a more restricted class than the graphs of small treewidth considered here.

## 3 Maximum-scoring path sets on series-parallel graphs

In this section, we first formally define our problem, review the known algorithm for the problem on sequences and show a simple new algorithm for computing it on directed acyclic series-parallel graphs. This algorithm is practical, as series-parallel graphs include graph classes used in pangenomics (see Section 3.3), but the algorithm also serves as a simple special case for the more complex algorithm for graphs of bounded treewidth described in the following sections.

### 3.1 Problem definition

Suppose we are given a directed graph *G* on vertices *v*_1_, …, *v*_*n*_, where each vertex *v*_*i*_ has weight *w*(*v*_*i*_) (either positive, negative, or 0), and a constant *c* ≥ 0. The score *w*(*π*) of path *π* consisting of vertices *u*_1_, *…*, *u*_*ℓ*_ is defined as $w\left(\pi \right)=-c+\sum_{j=1}^{\ell }w\left(u_{j}\right)$ . Constant *c* is thus a penalty for starting a path. Our goal is to find the *maximum-scoring path set* (MSPS), which is a set of vertex-disjoint (simple) paths {*π*_1_, *…*, *π*_*ℓ*_} with maximum total score *∑*_*i*_ *w*(*π*_*i*_). Note that in most of this paper we consider graph *G* to be directed, although one can also define the MSPS problem on undirected graphs.

The MSPS problem can be trivially proved to be NP-hard on general graphs by a reduction from the Hamiltonian path problem. Namely, a graph has a Hamiltonian path if and only if there is a solution to the MSPS problem with score at least *n* − *c* on the same graph with vertex weights all equal to 1 and penalty *c* such that 0 < *c* < *n*. This motivates our approach of studying the problem on special graph classes in order to obtain polynomial-time algorithms.

### 3.2 Relationship of the MSPS to maximum-scoring segment sets

As mentioned above, the MSPS is a direct generalization of the problem studied by Csűrös (2004) of finding maximum-scoring segment sets in a sequence of scores to graphs. In particular, when we consider graph *G* to be a simple path of vertices *v*_1_, …, *v*_*n*_, the MSPS task is to select segments of this path (or disjoint subpaths) that maximize the overall score. In this case, the task can be solved by a simple dynamic programming algorithm, which was introduced in Lemma 2 by Csűrös (2004).

Namely, consider subproblems *M*[*i*, *j*] for all 1 ≤ *i* ≤ *n* and *j* ∈ {0, 1} defined as the solution score of the MSPS problem on the subgraph defined by vertices *v*_1_, *…*, *v*_*i*_ with the additional constraint that:

• vertex *v*_*i*_ is used in one of the paths in the chosen path set if *j* = 1, and
• vertex *v*_*i*_ is not used in any of the chosen paths if *j* = 0.

Clearly, the score of the solution of the MSPS problem is simply max{*M*[*n*, 0], *M*[*n*, 1]}.

By definition, *M*[1, 0] = 0 and *M*[1, 1] = *w*(*v*_1_) − *c*. For all 1 < *i* ≤ *n*, the subproblems *M*[*i*, *j*] can be computed as:

$$\begin{array}{ll} M\left[i,0\right] & =\max\left\{M\left[i-1,0\right],M\left[i-1,1\right]\right\}\\ M\left[i,1\right] & =\max\left\{M\left[i-1,0\right]+w\left(v_{i}\right)-c,M\left[i-1,1\right]+w\left(v_{i}\right)\right\}. \end{array}$$

Note that when computing *M*[*i*, 0], we consider the case of starting a new path at *v*_*i*_ only when *v*_*i*−1_ was not used in a path. We can do this because if one path ends at *v*_*i*−1_ and another starts at *v*_*i*_, we can instead replace them with a single path and increase the score by *c* ≥ 0. This recurrence leads to an *O*(*n*)-time algorithm for solving the MSPS for path graphs.

### 3.3 Series-parallel graphs

To illustrate how to extend this algorithm to more general graphs, we now consider series-parallel graphs. These graphs take their name from their resemblance to electrical circuits, which can be connected in series or in parallel. In particular, we will consider *two-terminal series-parallel (TTSP) directed multigraphs* (Valdes et al., 1982). A single edge (*s*, *t*) with *s* ≠ *t* is a TTSP graph, and its endpoints *s* and *t* are called its *terminals*. Given two series-parallel graphs *G*_1_ and *G*_2_ whose terminals are *s*_1_ and *t*_1_ and *s*_2_ and *t*_2_, respectively, we can combine them into a larger series-parallel graph *G*_3_ either by merging *t*_1_ and *s*_2_ and taking *s*_1_ and *t*_2_ as *G*_3_’s terminals (*connecting* *G*_1_ *and* *G*_2_ *in series*) or by merging *s*_1_ and *s*_2_ into *s*_3_ and *t*_1_ and *t*_2_ into *t*_3_ and taking *s*_3_ and *t*_3_ as *G*_3_’s terminals (*connecting* *G*_1_ *and* *G*_2_ *in parallel*). Note that multiple edges between a pair of vertices may originate from parallel composition, but multiplicity of an edge makes no difference for our problem. Also note that TTSP directed graphs are acyclic, with all paths going in the direction from terminal *s* to terminal *t*.

**FIGURE 1:**
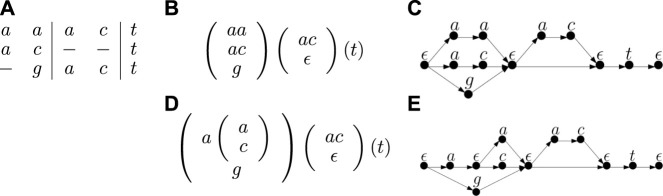
An example of a multiple sequence alignment with three sequences **(A)**, its representation as an EDS **(B)** and the corresponding TTSP graph **(C)**. The figure also shows an alternative representation of this alignment as a generalized EDS **(D)**, in which the first degenerate symbol has a nested degenerate symbol inside and the corresponding graph **(E)**.

TTSP graphs arise quite naturally in computational pangenomics. In particular, one commonly used pangenome representation is *elastic-degenerate strings* (EDSs), which are strings containing *elastic-degenerate symbols*. An elastic degenerate symbol is defined as a set of strings, potentially of different lengths. An EDS represents a set of strings; each string from this set is obtained by choosing one of the strings from each elastic degenerate symbol and concatenating them. We can easily convert an EDS into a graph in which each element of each elastic degenerate symbol is a separate path with one vertex per symbol (see Figure 1). These paths join at both ends in auxiliary vertices representing empty strings which are shared between successive pairs of symbols. The graphs obtained from EDSs by this transformation are a special case of the TTSP directed graphs. One can also further generalize EDSs by allowing nesting, where each string inside a degenerate symbol could be either a simple string or a set of strings written in the form of a generalized EDS. Such generalized EDSs were already considered by researchers in this area (N. Pisanti, personal communication) and can be captured by TTSP directed graphs.

**FIGURE 2:**
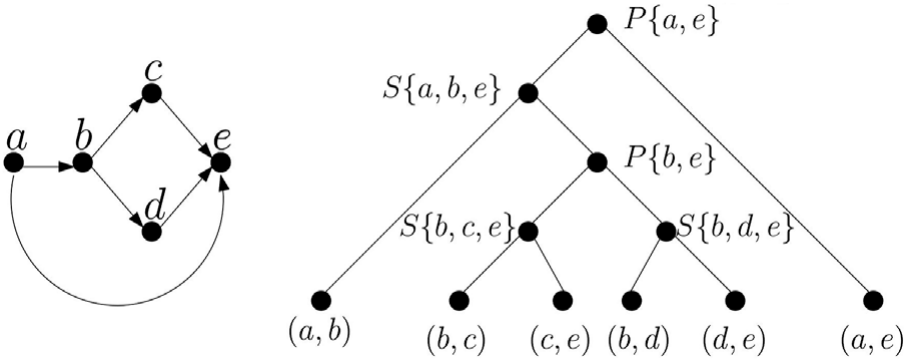
An example of TTSP directed graph and its decomposition tree. Internal nodes of the decomposition are labelled by character *S* or *P* denoting series and parallel composition, respectively and by a set that can be used as a bag in a tree decomposition (see Definition 1).

TTSP graphs can be represented by a *binary decomposition tree* (Valdes et al., 1982) with *G* at the root, the edges in *G* at the leaves, and a subgraph *H* of *G* at each internal node such that *H* is obtained by connecting the subgraphs *H*_1_ and *H*_2_ at its children by a series or parallel operation. We will call the subgraphs represented by nodes of the tree *modules*. An example of a TTSP directed graph and its tree decomposition is shown in Figure 2. Also note that the tree is not necessarily unique. TTSP graphs can be recognized in linear time and the decomposition tree can also be computed in linear time (Valdes et al., 1982), but in case of a graph built from an EDS or its generalization we can obtain the decomposition tree in a straightforward way while constructing the graph.

### 3.4 MSPS on series-parallel DAGs

Now, we consider the MSPS problem on TTSP directed graphs. We assume that graph *G* has already been decomposed into a tree of modules as outlined in the previous section. We will again employ dynamic programming. For each module *H*, we will compute four subproblems specifying if source *s* and sink *t* are used in the solution. Namely, let *M*[*H*, *u*_*s*_, *u*_*t*_] be the solution score of the MSPS problem for module *H*, where *u*_*s*_ is a binary value indicating if *s* is used and *u*_*t*_ is a binary variable indicating if *t* is used.

The computation proceeds from smaller modules towards the whole graph. Trivial modules consist of a single edge from *s* to *t* and their values are computed trivially (for *M*[*H*, 1, 1] it is optimal to connect *s* and *t* to a single path):

$$\begin{array}{c} M\left[H,0,0\right]=0\\ M\left[H,1,0\right]=w\left(s\right)-c\\ M\left[H,0,1\right]=w\left(t\right)-c\\ M\left[H,1,1\right]=w\left(s\right)+w\left(t\right)-c \end{array}$$

If module *H* is a series composition of modules *H*_1_ and *H*_2_ joined to module *H* by identifying source *s*_2_ with target *t*_1_ then

$$M\left[H,u_{s},u_{t}\right]=\max\left\{\begin{array}{c} M\left[H_{1},u_{s},0\right]+M\left[H_{2},0,u_{t}\right],\\ M\left[H_{1},u_{s},1\right]+M\left[H_{2},1,u_{t}\right]-\left(w\left(t_{1}\right)-c\right). \end{array}\right.$$

The first row corresponds to the case when *t*_1_ is not used in the solution. The second row corresponds to the case when it is used. Its weight and the path penalty were already accounted for in both subproblems, so they are subtracted to avoid double counting. Note that the case when a path starts or ends at *t*_1_ is accounted for by the second row of the formula as both subproblems *M*[*H*_1_, *u*_*s*_, 1] and *M*[*H*_2_, 1, *u*_*t*_] include the possibility of a solution in which *t*_1_ is a path of length 0. Of course, it is possible that the optimal solution of the corresponding subproblem will connect *t*_1_ to a longer path, but then such a longer path is also better than a path of length 0 as a part of the solution for *M*[*H*, *u*_*s*_, *u*_*t*_].

Similarly, if module *H* is a parallel composition of modules *H*_1_ and *H*_2_ joined to module *H* by identifying sources *s*_1_ and *s*_2_ to *s* and targets *t*_1_ and *t*_2_ to *t*, we can compute the corresponding subproblems as follows. First, if both *s* and *t* are unused in *H*, they must be unused in both *H*_1_ and *H*_2_:

$$M\left[H,0,0\right]=M\left[H_{1},0,0\right]+M\left[H_{2},0,0\right].$$

Second, if only vertex *s* is used, we will use the same version of the subproblem from one submodule, allowing a path to potentially continue from *s* in this module, but from the other submodule we take a solution which does not use *s* at all.

$$M\left[H,1,0\right]=\max\left\{\begin{array}{c} M\left[H_{1},1,0\right]+M\left[H_{2},0,0\right],\\ M\left[H_{1},0,0\right]+M\left[H_{2},1,0\right]. \end{array}\right.$$

Computation of *M*[*H*, 0, 1], where only *t* is used, is symmetrical. Finally, when both *s* and *t* are used in a path, we apply the same reasoning at both ends, thus considering four options.

$$M\left[H,1,1\right]=\max\left\{\begin{array}{c} M\left[H_{1},1,1\right]+M\left[H_{2},0,0\right],\\ M\left[H_{1},0,0\right]+M\left[H_{2},1,1\right],\\ M\left[H_{1},1,0\right]+M\left[H_{2},0,1\right],\\ M\left[H_{1},0,1\right]+M\left[H_{2},1,0\right]. \end{array}\right.$$

The above recurrences straightforwardly lead to a linear-time algorithm for computing the MSPS on series-parallel directed graphs.

### 3.5 MSPS on series-parallel undirected and cyclic graphs

**FIGURE 3:**
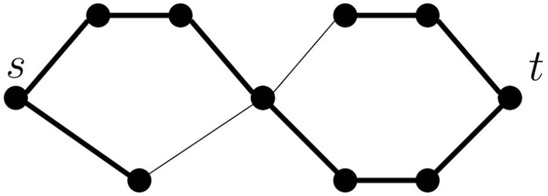
An example of a TTSP undirected graph and a single path forming a potential solution shown as thicker edges. Both the source *s* and target *t* are internal vertices of the path.

TTSP graphs are also studied in an undirected version, where the base case is an undirected edge connecting *s* and *t*, while the series and parallel composition operations remain the same. Although pangenomic graphs are more naturally represented as directed graphs, we note that our algorithm can be extended to the undirected TTSP graphs. It needs to consider more cases, because a terminal vertex (*s* or *t*) can be used inside a module either as an endpoint of a path or as a point in the middle of a path, and this restricts possible combinations with partial solutions in the other module (see Figure 3). In addition, if both *s* and *t* are endpoints of a path, we need to consider whether these are in fact ends of the same path or two separate paths. This helps us to avoid creating a cycle when attaching two modules in parallel. Although more complex, the algorithm for undirected case is fundamentally similar to the simple algorithm for TTSP directed graphs described above. Both algorithms use a modular decomposition of the graph and consider all combinations of necessary features of a partial solution in each module. As only a constant number of combinations is considered for each module, the running time remains linear. This leads us naturally to consider a more general graph decomposition, which we introduce in the following section. Our results there do not depend on graphs being directed or acyclic so, in particular, they apply to undirected series-parallel graphs.

## 4 Treewidth of a graph

The basic algorithm on paths from Section 3.2 can be generalized to directed trees. The case of a rooted directed tree with all edges directed consistently either from the root towards leaves or in the opposite direction can be solved quite easily in a bottom-up manner. However, trees themselves are not very useful as a pangenome representation. We therefore now extend the algorithm to a generalization of trees, namely, the graphs of a bounded treewidth, introduced by Robertson and Seymour (1986). In this section, we describe the treewidth parameter and its connections to pangenome graphs. In the next section, we provide the linear-time algorithm for MSPS on directed or undirected graphs of constant treewidth.

Definition 1 A tree decomposition of a graph *G* = (*V*, *E*) is a tree (X,I) where *I* is the set of tree edges and X is the set of tree vertices; each tree vertex $X_{i}\in X$ is a subset of *V*. The decomposition has to satisfy the following conditions:

1. *each vertex* *v* ∈ *V* *belongs to at least one* $X_{i}\in X$ ,
2. *for each edge* *e* ∈ *E* *, both its endpoints belong together to at least one* $X_{i}\in X$ ,
3. *if vertex* *v* ∈ *V* *belongs to both* *X*_*i*_ *and* *X*_*j*_ *, then* *v* *also belongs to each* *X*_*k*_ *on the unique path between* *X*_*i*_ *and* *X*_*j*_ *in the tree* (X,I) .

**FIGURE 4:**
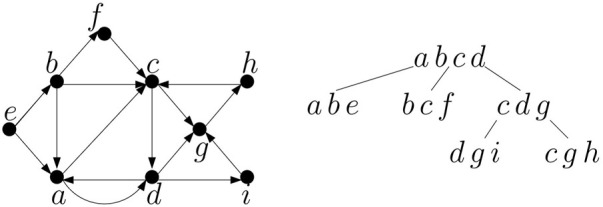
A graph and its tree decomposition of width 3.

To avoid confusion between vertices of *G* and vertices of the decomposition tree, we will call tree vertices $X_{i}\in X$ *bags*. The *width* of a tree decomposition (X,I) is $\max_{X_{i}\in X}|X_{i}|-1$ and the *treewidth of a graph* *G* is the minimum width over all tree decompositions of *G*. An example of a graph and its tree decomposition is shown in Figure 4. A *path decomposition* is a special case of a tree decomposition where tree (X,I) is a single path of bags. The *pathwidth of a graph* is then the minimum width over all path decompositions.

Many NP-hard problems can be solved efficiently on graphs with constant treewidth (Arnborg et al., 1991; Bodlaender, 1997), if a tree decomposition of a small width is provided as a part of the input. The problem of determining a treewidth of a graph is NP-hard (Arnborg et al., 1987), but for a constant *k*, it is possible to find a tree decomposition of width at most *k* (if it exists) in linear time (Bodlaender, 1996).

It is easy to see that a tree (or a forest) has treewidth 1, because we can create a decomposition in which each bag contains endpoints of a single edge. Series-parallel graphs have treewidth at most 2 (Brandstädt et al., 1999), and their tree decomposition closely mirrors the decomposition into modules described in Section 3.3. Namely, we create a bag for each node of the decomposition tree. For leaves, the bag will contain endpoints of a single edge. For internal nodes, the bag will contain the sources and sinks of both submodules, and thus will have size 2 for parallel composition and 3 for serial composition (see Figure 2).

Tomescu et al. (2015) introduce the notion of arc-width which is the smallest number of (possibly overlapping) paths needed to cover all edges of a directed graph. For simplicity, we will assume that the paths cover also all vertices of the graph, which is automatically true in all graphs without isolated vertices. The arc-width of a DAG can be computed in polynomial time (Tomescu et al., 2015). If the arc-width is constant, it can be even found in time linear in the size of the graph by subdividing each edge by a new vertex and using recent parameterized algorithms for minimum path cover (Cáceres et al., 2022). Note that if we have a pangenome in the form of a DAG constructed from a multiple sequence alignment of *k* sequences, it will typically have arc-width at most *k*, because each sequence in the alignment corresponds to some path in the graph and each edge of the graph belongs to one of these paths. If the sequences in the pangenome have a lot of shared parts, the arc-width may be even lower than *k*. It is reasonable to assume that the paths corresponding to individual sequences will be stored when constructing the pangenome graph. This leads us to the following decomposition algorithm for such graphs.

Lemma 1 Any DAG *G* with arc-width *k* has both pathwidth and treewidth at most *k* and the corresponding path decomposition can be computed in *O*(*nk*) time provided the *k* paths covering all edges are given.

Proof. Let *v*_1_, *…*, *v*_*n*_ be the vertices of *G* in a topological order, which is an order such that for each edge (*v*_*i*_, *v*_*j*_) we have *i* < *j*. A topological order can be computed in time linear in the size of the graph, and as our graph has at most *nk* edges, the running time is *O*(*nk*). Each of the *k* input paths is a subsequence of this topological order.

For each vertex *v*_*i*_ we create a bag *X*_*i*_ that contains *v*_*i*_ and for each input path *π* it also contains the last vertex among *v*_1_, …*v*_*i*−1_ that belongs to *π* (if any). To form the path, we connect bags in the order *X*_1_, *X*_2_, *…*, *X*_*n*_. The size of each bag is at most *k* + 1, and we will prove that they satisfy all conditions from Definition 1. Clearly, the bags cover all vertices of *G*. Each edge (*v*_*j*_, *v*_*i*_) is covered by some path, and therefore *v*_*j*_ will be in bag *X*_*i*_ due to *v*_*j*_ being the last vertex on this particular path before *v*_*i*_. Finally, consider bags containing some vertex *v*_*i*_. The first of them is *X*_*i*_. Vertex *v*_*i*_ will be in *X*_*j*_ for *j* ≥ *i* as long as at least one path passing through *v*_*i*_ does not contain any of the vertices *v*_*i*+1_, *…*, *v*_*j*−1_. This is true up to some *X*_*k*_, and thus bags containing *v*_*i*_ form a subpath starting at *X*_*i*_ and ending at *X*_*k*_, which implies the third condition of Definition 1.

Bags can be formed by a simple sweep along all paths simultaneously, at vertex *v*_*i*_ advancing a pointer on all paths containing it. This can be done trivially in *O*(*nk*) time, where *k* is the number of paths.

## 5 MSPS on graphs of constant treewidth

In this section, we show our algorithm that computes MSPS on a graph of constant treewidth *k*, provided it is given a tree decomposition of width *k* on input. As is often the case for algorithms on graphs with constant treewidth, the running time will be linear in *n*, but superexponential in *k*. We will consider primarily directed graphs but we note throughout the text how it can be adapted to undirected graphs. Note that we do not require directed graphs to be acyclic. For simplicity, we will assume that the graph does not contain self-loops or parallel edges, as these are not needed to obtain an optimal solution of the MSPS problem.

### 5.1 Preliminary observations and preprocessing

**FIGURE 5:**
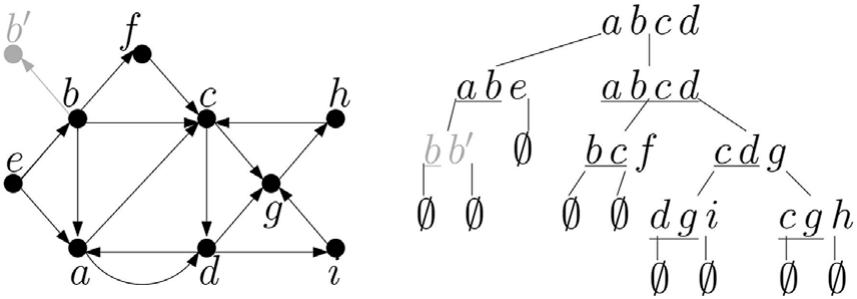
The graph and its tree decomposition from Figure 4 after preprocessing, assuming that *b* was the only vertex with weight greater than penalty *c*. Note that bag {*a*, *b*, *c*, *d*} originally had three children, and thus it was duplicated during preprocessing to obtain a binary tree. Terminals are underlined in each bag.

First we will do several preprocessing steps to convert the input graph and its tree decomposition to a form more convenient for our algorithm, and we will solve a slightly modified version of the problem on this preprocessed graph, but the result can be converted to the optimal solution of the original problem on the original graph. Namely, we will consider a variant of the MSPS problem where the solution may contain a mix of paths and cycles (each path or cycle uses each vertex at most once). This is equivalent to the original problem, as each cycle can be changed into a path by omitting any one of its edges without changing the score. Further, we will allow only non-trivial paths (or cycles) with at least one edge in the solution. This disallows solutions where a vertex of weight at least *c* is considered as a separate path of length 0. However, we can modify the original graph by creating an auxiliary vertex *v*′ with weight 0 for each original vertex *v* with weight at least *c* and connecting the pair (*v*, *v*′) by an edge. This change does not change the treewidth of the graph, and the tree decomposition can be extended by a new leaf bag {*v*, *v*′} connected to one of the bags containing *v* (see Figure 5). A solution containing edge (*v*, *v*′) in the preprocessed graph will be converted to contain *v* alone in the final answer (this is possible as there is no outgoing edge from *v*′ and thus a path must end there).

After these changes, we can characterize a potential solution of the problem purely as a set of edges such that each vertex is incident to at most one incoming and at most one outgoing edge (or, for undirected graphs, at most two edges in total). The score of a solution is the sum of weights of vertices that are incident to at least one edge minus penalty *c* for each connected component in the subgraph formed by these edges.

Consider a tree decomposition rooted in an arbitrary bag. Without loss of generality we can assume that the tree of the decomposition is binary, that is, each bag has at most two children. If bag *X* has more than two children, we can make additional copies of the bag, connect them to a binary tree, and place the children of the original *X* to the leaves of this new tree structure, which will replace *X*. In addition, we will add auxiliary leaves containing empty bags so that each nonempty bag of the decomposition has exactly two children (see Figure 5). These changes will not asymptotically increase the number of bags, because the number of new empty bags will be at most twice the number of the original bags and the number of bags added to replace high-degree nodes is less than the number of the original bags (for a bag with *k* children we add *k* − 2 new bags). The two children of any internal node of the tree will be arbitrarily labeled as left and right.

Finally, the definition of a tree decomposition guarantees that for each edge there is at least one bag where both its endpoints co-occur, but there could be multiple such bags. For the purpose of our algorithm, we will assign each edge to exactly one of those bags. Each bag thus can be considered as a set of vertices and edges between those vertices.

Consider a bag *X* with bag *Y* as its parent in the tree decomposition. The vertices in *X* ∩ *Y* will be called *terminals* of *X*, in analogy with terminals of the parallel-series graphs. By the third property of tree decomposition, non-terminal vertices of *X* occur only in the subtree rooted at *X*, while terminals are the vertices which may be connected to the rest of the graph. In the root, we can consider an empty set of terminals (as if the parent of the root was another auxiliary empty bag).

### 5.2 Algorithm overview

We will process the bags of the decomposition bottom-up and for each bag *X* compute quantity *M*[*X*, *C*], where *C* is a *configuration* determining the behaviour of the solution paths at the terminals of *X*. Value *M*[*X*, *C*] is the best solution considering only edges in the bags of the subtree rooted at *X* and obeying constraints in configuration *C*.

A configuration for bag *X* with the set of terminals *T* is specified as follows. Each terminal *v* has at most two incident edges in the solution; we will therefore consider two slots for edges per terminal. In directed graphs, one slot is for an incoming edge and one for an outgoing edge. The slot for an edge consists of the following:

(a) Information whether the slot is used or not (for example, in the slot for incoming edge of *v* we specify whether the solution contains an edge ending in *v*).
(b) If the slot is used, let us follow (possibly backwards) the path or cycle from *v* along this edge and let *u* be the first vertex from set *T* we meet. We store this vertex *u* or an indicator that such a vertex does not exist. We will say that this slot *points* to *u*.

**FIGURE 6:**
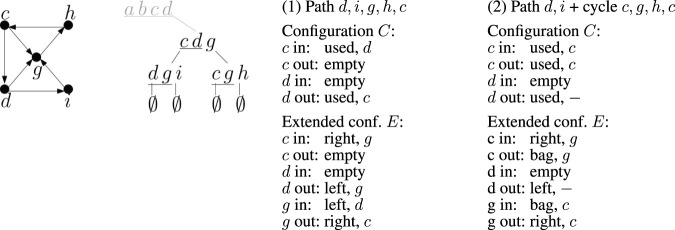
An example of two partial solutions (1) and (2) and their corresponding configurations and extended configurations when processing bag *X* = {*c*, *d*, *g*} in the tree decomposition from Figure 5. The relevant part of the decomposition tree is shown here. Assuming that each edge is placed to the highest bag containing both of its endpoints, the subtree rooted in *X* contains all edges shown in the graph on the left, except for edge (*c*, *d*), which is considered in the root bag. Bag *X* contains edges (*c*, *g*), (*d*, *g*). For each configuration and extended configuration we show the values stored in incoming and outgoing slots.

Each slot thus has |*T*| + 2 possible values: all terminals and two special indicators for an empty slot and the last vertex from *T*. There are 2|*T*| slots, leading to an upper bound of (|*T*| + 2)^2|*T*|^ on the number of configurations. Two example configurations are shown in Figure 6.

The algorithm starts in the leaves of the tree, which have empty auxiliary bags, and thus they have a single empty configuration *C*, and *M*[*X*, *C*] = 0. The algorithm then proceeds towards the root. In the root, we have again only a single configuration, and the value *M*[*X*, *C*] for this configuration will be the optimal score for the whole graph.

Consider now bag *X* with the set of terminals *T*, which forms an internal node of the decomposition tree with children *X*_1_ and *X*_2_. To compute values *M*[*X*, *C*], we will iterate over a bigger set of more detailed configurations, which we call *extended configurations*. An extended configuration has two slots for every vertex of *X* (not only the terminals) and for each slot it specifies one of 3|*X*| + 4 possibilities as follows:

(a) Information whether the edge occupying the slot belongs to the left subtree of the decomposition, the right subtree, bag *X* or slot is unused (4 options in total).
(b) If the slot is used, let us follow the path from the current vertex along this edge and let *u* be the first vertex from *X* we meet. We store this vertex *u* or an indicator that such a vertex does not exist (in total, |*X*| + 1 possible values if one the first three options is used above). We will say that this slot *points* to *u* *via* left subtree, via right subtree or via *X*, depending on the indicator in part (a).

The number of extended configurations is upper-bounded by (3|*X*| + 4)^2|*X*|^. However, some extended configurations are not valid and thus do not need to be considered by the algorithm; we describe the subroutine is_valid for determining if an extended configuration is valid in the next subsection. In undirected graphs, some extended configurations are equivalent as the two slots for each vertex are symmetrical.

For each extended configuration *E* we will compute the optimal score *M*′[*X*, *E*] over solutions within the current subtree that obey constraints given by *E*. There is only one configuration *C*_*i*_ in each child *X*_*i*_ which is consistent with *E*, and this *C*_*i*_ can be easily computed using subroutine child_conf below. We can therefore add up pre-computed scores *M*[*X*_*i*_, *C*_*i*_] for both children of *X*. However, we need to further add or subtract vertex weights and penalties to account for the overall solution. Namely, we add the weights of all vertices which are adjacent to at least one edge in bag *X* but are not adjacent to any edge in the subtrees rooted at *X*_1_ and *X*_2_. These vertices can be easily determined from the extended configuration *E*. To avoid double counting, we subtract the weights of vertices which were used in both subtrees. Finally, we compute how many penalties we need to add or subtract by checking how many paths from subtrees are now connected to bigger components and how many new paths or cycles are added. This is done using subroutine penalties below.

Each *M*[*X*, *C*] is the maximum of *M*′[*X*, *E*] over extended configurations *E* which are consistent with *C*. For each *E* we can easily compute its unique consistent *C* using subroutine reduce_conf below. If a configuration *C* is not consistent with any valid extended configuration *E*, we will consider its score *M*[*X*, *C*] to be −*∞*. Table *M*′ does not need to be stored explicitly, but each computed value of *M*′ can be directly used to update the corresponding entry of *M*. For tracing back the actual paths, we would store for each configuration *C* also the best consistent extended configuration *E* from which its score was copied. This extended configuration can be used to infer configurations of the children (using subroutine child_conf) as well as edges of *X* used in the solution (these are stored in individual slots of *E* as pointers to another vertex via the current bag *X*).

### 5.3 Additional details

In this section, we describe the four subroutines introduced in the algorithm overview.

Subroutine is_valid: Given an extended configuration *E* of bag *X*, we want to check whether this configuration is valid, meaning that it does not impose contradictory constraints on the solution and correctly uses edges of *X*. Namely, for all vertices of *X* we will check the following. If one of the slots of some vertex *v* ∈ *X* points to vertex *u* via subtree *X*_*i*_, then also *u* has to point to *v* via the same subtree and both *u* and *v* must belong to this subtree. To prove that this must be the case in a valid configuration, consider the path from *u* to *v* (the case when the path is directed from *v* to *u* is symmetrical). If this path consists of a single edge, the claim clearly holds. Otherwise there are some intervening vertices on the path. These vertices do not belong to *X*, because *v* is defined as the first vertex from *X* on the path. All these vertices must be from the subtree rooted at *X*_*i*_, because the edge leaving *u* is from this subtree and this subtree is connected to the rest of the decomposition tree only through bag *X*. This implies that the edge entering *v* is also from this subtree, which is what we needed to prove.

Similarly, *u* and *v* can also point to each other via an edge from *X*, and such an edge has to exist in bag *X*. Since we also allow cycles, vertex *v* can point to vertex *u* twice, and *u* must then also point to *v* twice. For directed graphs these reciprocal pointers must have a correct orientation; for undirected graphs we consider the two slots as equivalent and we must check if they can be correctly paired. Note that even some valid extended configurations will have the score of −*∞*, because the set of vertex connections specified in the configuration may be unachievable using the edges of the graph.

Subroutine reduce_conf: The goal of this step is to take an extended configuration *E* of bag *X* and compute its corresponding configuration *C*. We will take all terminals and their edge slots and reduce their stored values from four options to the less detailed two options used in a configurations. If the slot is used, *E* also points to the next vertex from *X* on the path. In *C*, we need to point to the first vertex from a potentially smaller set *T* of terminals, which is found by following these pointers in *E* until we arrive at a terminal. Note that to follow the pointers, we have to determine in each vertex, which of its two slots points back to the vertex we came from and which points forward.

Subroutine child_conf: The goal of this step is to take an extended configuration *E* of bag *X* and to compute the configuration *C*_*i*_ of its child *X*_*i*_ consistent with *E*. Let *T*_*i*_ be the set of all terminals of *X*_*i*_. Note that *T*_*i*_ ⊆ *X*. Configuration *E* specifies for each slot whether this slot is occupied by an edge in subtree *X*_*i*_, which is the binary option needed in *C*_*i*_. If the slot is thus occupied, the pointer will point to the first vertex of *X* on the path, which is in this case also the first vertex of *T*_*i*_, and so we store it in *C*_*i*_.

Subroutine penalties: We are given an extended configuration *E* of bag *X*, and we want to compute how many penalties *c* should be added or subtracted. Some connected components in the solution specified by *M*′[*X*, *E*] do not contain any vertices from *X*. These are completely contained in one of the subtrees of *X* and are accounted for there. We can easily count the remaining components by following the pointers connecting successive vertices of *X* on the paths or cycles. We then subtract value *c* from the score for each found component. Next, we take the configurations *C*_1_ and *C*_2_ consistent with *E* and corresponding to the two children *X*_1_ and *X*_2_ of bag *X*. Following the pointers stored in each *C*_*i*_, we can again count the number of components that use at least one of the terminals of bag *X*_*i*_ within the partial solution for the respective subtree. We add back penalty *c* for each such component, as it was considered when examining *E* either as a separate component or as a part of a bigger component containing also paths from the other subtree and/or edges of bag *X*.

### 5.4 Time and space complexity

We are given a graph with *n* vertices and its tree decomposition of width *w*. We will assume that the decomposition has *O*(*n*) bags, which is always possible to achieve (Arnborg et al., 1991). Note that the number of edges of a graph with treewidth *w* is at most *wn* (Arnborg et al., 1991). Graph preprocessing can be done in *O*(*wn*) time and the number of bags will remain *O*(*n*). To allow checking if vertices and edges belong to particular bags and to convert from a graph-wide vertex number to a vertex number used within a bag, we can build appropriate hash tables during graph preprocessing. Let *f*(*b*) be an upper bound on the number of the extended configurations for a bag with *b* vertices. We proved above that *f*(*b*) ≤ (3*b* + 4)^2*b*^. The algorithm iterates through these extended configurations and for each makes a constant number of calls to the subroutines described in the previous section. These subroutines do mostly trivial operations on configurations, and each runs in *O*(*b*) time. This includes subroutine penalties which needs to find connected components in a graph with *O*(*b*) vertices and edges formed by slot pointers. Thus given a graph with *n* vertices and its tree decomposition with treewidth *w* and *O*(*n*) bags, the running time of the algorithm is *O*(*w* ⋅ *f* (*w* + 1) ⋅ *n*), which is *O*(*n*) if *w* is a constant. For space, let *g* (*t*) be an upper bound on the number of configurations for a bag with *b* vertices and *t* terminals; we proved that *g*(*t*) ≤ (*t* + 2)^2*t*^. For each configuration, we store its score and the optimal extended configuration in space of size *O*(*b*). As both *b* and *t* are upper-bounded by *w* + 1, the overall space for a decomposition with treewidth *w* and *O*(*n*) bags is *O*(*w* ⋅ *g* (*w* + 1) ⋅ *n*), which is also *O*(*n*) for constant *w*. Note that a careful implementation could avoid iterating through many invalid configurations and extended configurations, thus significantly reducing the superexponential upper bounds *f*(*w* + 1) and *g*(*w* + 1).

## 6 Conclusion

In this paper, we have introduced the *maximum-scoring path sets problem* (MSPS) which generalizes previously established maximum-scoring segment sets problem from sequences to graphs. The maximum-scoring segment sets problem has been shown to encompass many practical problems in analysis of biological sequences (Csűrös, 2004), and our new MSPS problem directly generalizes these applications to pangenome graphs.

We have also provided a general algorithm for solving MSPS with time complexity linear in the size of the graph but superexponential in the treewidth parameter of the graph. The algorithm is applicable to both directed and undirected graphs, including graphs with cycles, as long as the treewidth is small. We note that some established representations of pangenome graphs (including elastic degenerate strings and directed acyclic graphs with a constant arc-width) indeed have a small treewidth parameter which makes the application of our algorithm practical in these cases.

There are multiple avenues for future work in this area. First, at present there is no single universally accepted method for transforming a set of sequences or a multiple alignment to a pangenome graph. Indeed, the same set of sequences can be potentially represented by many different graphs with quite different properties. The running time of downstream analysis algorithms is influenced by some of these properties, including the treewidth considered in our work as well as other parameters considered previously (Tomescu et al., 2015; Mäkinen et al., 2019; Chang et al., 2020; Chandra and Jain, 2023). To this end, it would be interesting to conduct a practical comparison of treewidth and other relevant parameters in pangenome graphs constructed by currently available software tools from real biological sequences and study whether modifications in construction algorithms lead to more favorable values of such parameters.

Even if a pangenome graph as a whole does not have a small treewidth but only a few bags are large, with some modifications our algorithm may still be applicable. Parts of the computation corresponding to the large bags may be either replaced by heuristics reducing the set of configurations considered, or replaced by methods such as integer linear programming. With such approaches it should be possible to develop a practical software tool applicable to many instances of the real pangenome graphs.

Our generalization of the maximum-scoring segments sets replaced segments with paths, which seems to be a natural extension, as in acyclic pangenomes the original genome sequences indeed correspond to paths. However, in a pangenome with cycles, a sequence with repeated substrings can be represented by a walk with some vertices repeating multiple times. It might be therefore desirable to consider versions of the problem allowing such walks, but the problem needs to be formulated with care to avoid unwanted optima in which a cycle with a positive score is repeated infinitely many times. In diploid organisms, a typical chromosome is present in two similar copies, which motivates further extension of looking for two separate sets of disjoint paths or walks, one per haploid genome. An appropriate scoring of such solution sets to avoid trivial outcomes is also a challenge.

## Data Availability

The original contributions presented in the study are included in the article/supplementary material, further inquiries can be directed to the corresponding author.
